# A case of severe benzalkonium chloride intoxication in a cat

**DOI:** 10.1186/s13028-024-00737-x

**Published:** 2024-04-15

**Authors:** Silva Rubini, Roberto Rubini, Silvia Bertocchi, Silvia Zordan, Alice Magri, Filippo Barsi, Maria Sampieri, Carlo Alessandro Locatelli, Erika Baldini, Stefano Manfredini, Silvia Vertuani

**Affiliations:** 1 Zooprophylactic Institute of Lombardy and Emilia Romagna, Ferrara Territorial Office, Via Modena 483, 44124 Ferrara, Italy; 2 Europa Veterinary Clinic, Via Arginone 381/C, 44124 Ferrara, Italy; 3 Zooprophylactic Institute of Lombardy and Emilia Romagna, Bologna Territorial Office, Via P. Fiorini 5, 40127 Bologna, Italy; 4 Poison Control Center of Pavia-National Center for Toxicological Information-Clinical and Experimental Toxicology Laboratories, Clinical Scientific Institutes Maugeri SpA SB, IRCCS Pavia, Via Salvatore Maugeri 10, 27100 Pavia, Italy; 5 Department of Life Sciences and Biotechnology, Faculty of Medicine, Pharmacy and Prevention, University of Ferrara, Via Luigi Borsari 46, 44121 Ferrara, Italy

**Keywords:** BAC, Benzalkonium chloride, Feline, Oral ulcerations, QAC, Toxidrome

## Abstract

**Background:**

Benzalkonium chloride (BAC) is a quaternary ammonium compound (QAC), that can be found in a wide variety of household products–from disinfectants to medicaments and home fragrances–but also professional products. In pets, cats have long been reported as more sensitive than dogs to QACs; in fact, signs of irritation such as oral ulcerations, stomatitis and pharyngitis can be observed after contact with concentrations of 2% or lower. In a review of 245 cases of BAC exposure in cats, reported by the Veterinary Poisons Information Service (United Kingdom) only 1.2% of the cases died or were euthanized. Nevertheless, BAC toxidromes in cats can result in transitory CNS and respiratory distress, as well as severe mucosal and cutaneous lesions. Currently, only a few reports are available concerning BAC poisoning in this species.

**Case presentation:**

A 4 month-old kitten presented with severe glossitis, lameness in the hindlimbs and episodes of vomiting and diarrhoea. The cause was unknown until the owners reported use of a BAC-containing mould remover (5%) 4 days later. The patient developed severe oral burns requiring a pharyngeal tube for feeding and severe cutaneous chemical burns. The kitten was managed with supportive therapy and required hospitalization for 10 days. The symptoms disappeared completely 3 weeks after exposure.

**Conclusions:**

BAC is a very common compound contained in several household and professional products but, to the best of our knowledge, no previous case had been reported in Italy. We hope that this report will help raise awareness on the hazards of BAC products for cats in both domestic and work contexts.

## Background

**Fig. 1:**
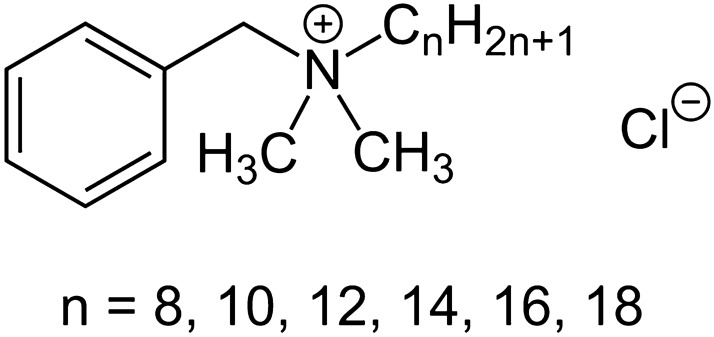
BAC chemical structure, alkyl dimethyl benzyl ammonium chloride, CAS number 68424–85-1 https://commons.wikimedia.org/w/index.php?curid=29727055

Benzalkonium chloride (BAC, alkyldimethylbenzylammonium chloride, CAS number 68424-85-1) (Fig. 1) is a quaternary ammonium compound (QAC), classified as a cationic surfactant detergent. QACs, including BAC, are biocidal against bacteria, some fungi and yeast; they are not biocidal against bacterial spores. They interact with cell membranes or transmembrane proteins by inserting their hydrophobic alkyl tail into the hydrophobic membrane core to destabilize the membrane [1].

BAC is a common compound in disinfectants, preservatives, medicaments and household sanitizers [2]. The BAC toxidrome is dose-dependent and can be divided into local effects and systemic effects [3]. The effects most frequently observed in the veterinarian practice are local and associated with irritation of the exposed tissues and can manifest as oral ulcerations, stomatitis pharyngitis, hair loss and chemical burns [4–6]. Systemic effects are less common; however, in case of severe toxicosis, dehydration, CNS and respiratory alterations, hematemesis, seizures, collapse and coma may be observed [4–7]. Pharmaceutical or cosmetic products for dermal or ocular use in humans generally contain 0.004 to 0.01% BAC, while concentrations above 0.1% (or 1 g/l) are considered potentially caustic and corrosive [8]. BAC and QACs can also be used in the agricultural sector, for the disinfection of buildings or equipment, but not on agricultural crops, where they are prohibited. Therefore, these compounds can be found in agricultural storage areas frequented by pets. In pets, cats have been reported as more sensitive than dogs to QACs; indeed, oral ulcerations, stomatitis and pharyngitis can be observed in concentrations of 2% or lower [9]. Since BAC is also a common component of certain medicaments, cats can also develop oral and oesophageal ulcerations as consequences of self-grooming if the skin has been treated with undiluted disinfectants [10].

## Case presentation

**Fig. 2:**
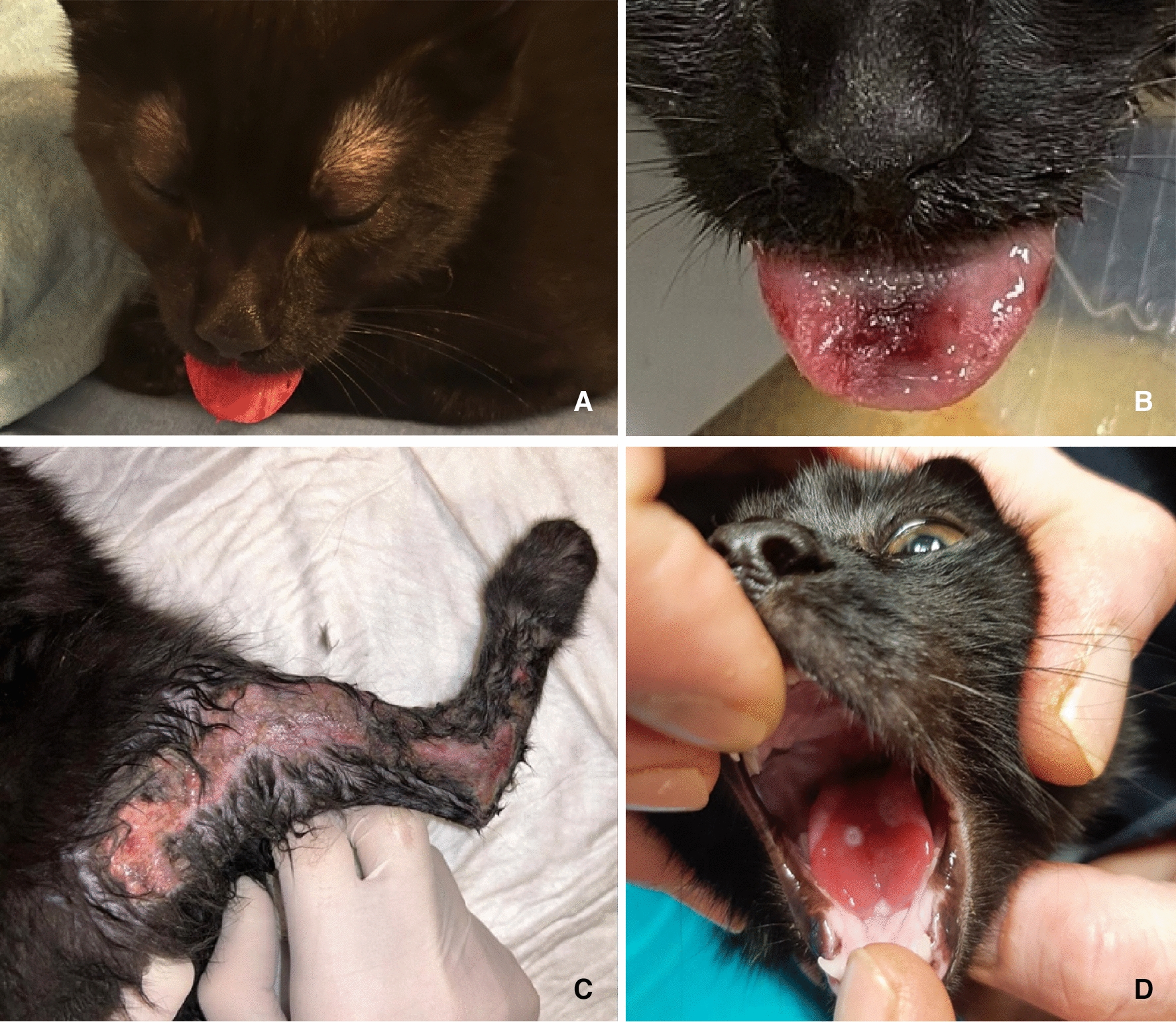
Lesions observed on the patient. **A** Enlargement of the tongue. **B** Chemical burns observed on the tongue. **C** Cutaneous irritation and excessive grooming of the hindlimb. **D** Chemical burns on the tongue and palate

A 4-month-old male European shorthair kitten was brought to the Europa Veterinary Clinic (Ferrara, Italy) due to severe enlargement of the tongue, which prevented the mouth from closing (Fig. 2a). The owners also reported state of anxiety, lameness in the hindlimbs, and episodes of vomiting and diarrhoea during the night.

The kitten at the time of admission weighed 2 kg. During the physical examination, the kitten showed the following physiological vital parameters: rectal temperature of 38 °C and regular pulse; no alteration of the cardiopulmonary auscultation and explorable lymph nodes was observed. As mentioned above, inspection of the buccal area revealed enlargement of the tongue, due to severe oedema and inflammation, and sialorrhea. The tongue had reduced mobility and showed signs of a tongue ulceration, also observed in the pharynx (Fig. 2b). No foreign body was visible in the buccal cavity or in the pharynx.

The veterinary staff suggested a brief admission and initiated therapy with a single dose of prednisolone 1 mg/kg, and analgesic treatment with methadone 0.1 mg/kg IM every 5 h. In the evening, the symptoms got worse: the body temperature rose to 40 °C and there was no sign of improvement in the tongue. The kitten was therefore hospitalized and started on antibiotic therapy with cefazolin 15 mg/kg EV BID.

The following day, the patient still did not show any improvement; furthermore, he could not eat independently and forced feeding caused discomfort and pain. In view of this, the kitten was sedated to insert a pharyngo-gastric feeding tube. During sedation, the mouth and the pharynx were further inspected, and no foreign body was found. Also, wet fur caused by excessive grooming of the posterior limb was observed.

Due to the persistent hyperthermia, meloxicam 0.05 mg/kg SC SID was administered. 48 h after hospitalization, the patient developed a new exudative cutaneous and subcutaneous erythema on the hindlimb, corresponding to the area of excessive grooming observed during the sedation (Fig. 2c). The lesion was painful and hyperaemic.

Four days after the clinical consultation, the owners reported the use of an antifungal substance including in areas of the house frequented by the cat; the active ingredient of this product was BAC at a concentration of 5%. The product was applied once, according to the manufacturer's instructions; however, a small quantity accidentally spilled onto the floor, which the kitten later stepped in. The kitten was exposed to the product for a few hours. Upon examination, the veterinarians did not detect any significant odour that could be ascribed to the BAC product. Due to the stable vital parameters, the veterinary staff agreed to a partial discharge of the patient, who was brought to the clinic for daily medication in the following seven days.

On the 6 day the patient finally showed signs of improvement, with a reduction in the lingual oedema. Despite this, chemical burns were noticed on the palate, tongue and hindlimbs (Fig. 2d). The chemical burns on the hindlimbs were treated by frequent washing with sterile deionized water and medicated gauze with 2 mg hyaluronic acid. The chemical burns in the mouth and on chin and tail were instead treated with frequent washing with sterile deionized water. The oral chemical burns were also medicated with protective and soothing oral mucosal gels.

Symptoms progressively improved and the chemical burns healed centripetally, i.e. healing started from the outer edges and progressed towards the centre. Centripetal healing is often a good sign, indicating that the immune system and repair mechanisms are effectively working to close a wound or clear an infection or inflammation from the skin. Indeed, the patient was able to chew and swallow without loss of food from the mouth. Considering the gradual improvements, the pharyngo-gastric tube was removed 9 days after the first consultation.

Three weeks after the poisoning, the cat showed weight gain and complete healing of the cutaneous chemical burns.

## Discussion and conclusions

QACs, including BAC, are found in a wide variety of household products, from disinfectants to medicaments and home fragrances. Proportionate to the concentration, they can cause local or systemic symptoms. Cats have long been known to be susceptible to BAC, even at concentrations of 1.5–2% [9]. Although few reports are available on BAC poisoning in this species, a number of cases have been reported in the annual VPIS reports. In any case, it is important to note that in Italy this is the first reported case.

Early reports concern the exposure of cats in a laboratory facility to concentrated disinfectant solutions containing BAC at 20% [11]. Subjects showed anorexia, hypersalivation, depression, dehydration, ulcerations, and nasal and ocular discharge. This was further investigated in a laboratory experiment by applying 0.5 mL of the disinfectant to the paw of a cat, which was euthanized on the 4 day after the contact, because of the damage caused by the poisoning. A histopathological examination revealed ulceration of the oral epithelium and tongue that extended to the muscular layer and necrotic epithelium on the exposed submucosa of the proximal oesophagus, and bronchopneumonia [11]. Toxicosis in three cats was also reported as consequence of using undiluted conductive gel containing 17% BAC during an ECG examination [12]. Patients developed severe oral ulceration after grooming, and in one case oesophagitis and oesophageal stricture were also observed [12].

More recently, a retrospective study was performed on data concerning 245 cats referred to the Veterinary Poisons Information Service (VPIS-UK) over a period of 15 years [7]. In these patients, poisoning occurred after ingestion and/or contact with household cleaners. The most commonly observed signs were hypersalivation (53.9%), tongue ulceration (40.4%), hyperthermia (40.4%) and oral ulceration (22.9%), followed by symptoms associated with CNS depressions. Patients were treated with antibiotics (82%), fluids (50.2%), analgesia (45.3%), gastroprotectants (31%), dermal decontamination (24.1%) and steroids (22.7%). The outcome was fatal for 1.2% of the patients, who showed severe respiratory symptoms, although the authors state that the significance of this is uncertain due to the small number of fatal cases reported [7].

A retrospective study was also conducted by the Department of Small Animal Clinical Sciences (East Lansing, Michigan), which analysed the poisoning of 6 cats (three males and three females) over a period of 7 years [13]. All cases concerned poisoning after contact with or ingestion of potpourri oil; this can be toxic for cats due to it containing QACs used as stabilizers and essential oils [9]. The subjects had a median age of 2.15 years and were referred to the hospital an average of 10.5 h after the suspected ingestion. Physical examination revealed in all patients: hyperthermia, tachypnoea and severe oral and lingual ulcerations; depression and ptyalism were observed in four patients, increased lung sounds and oil staining of the haircoat in two, and heart murmur in one. Four patients received gastrointestinal protectants and buprenorphine during hospitalization. Additionally, the patients were medicated with oral or dermal rinsing with chlorhexidine-based solutions. All the cats survived the poisoning [13].

In this case study, we report the BAC intoxication of a 4 month-old kitten. Although BAC is a very common compound, to the best of our knowledge, no previous cases have been reported in Italy. BAC poisoning can also occur by accidental contact with concentrated solutions that may be stored among agricultural equipment for disinfectant use, following accidental breakage and/or leakage from the container. In this case, pets can suffer much more severe exposure. In all likelihood, the cat in our study stepped in the solution that spilled on the floor and ingested the BAC while licking to clean itself. It is difficult to imagine otherwise such severe poisoning through contact with solutions found among household products. The patient developed chemical burns on the hindlimbs, on the palate and on the tongue but did not suffer any respiratory distress. The therapy and support established by the veterinary staff were focused on symptomatic treatment and prevention of secondary infections. This was also due to the late report of the owners on the use of a BAC-based product in the domestic environment and the non-specific symptoms that the patient displayed at the first consultation. However, management using analgesia, steroids, rehydration and gastroprotection have been reported as successful in most of the cases of BAC poisoning [7, 13]. Emesis is not recommended because the quantity of BAC ingested is usually small, and there is the potential risk of aspiration, which could worsen the respiratory symptoms. Antibiotics may be considered in the treatment of BAC poisoning, mostly to treat secondary infections; however, the use of antibiotics must be carefully evaluated [7].

As stated above, BAC is a common compound in various household and professional products, ranging from disinfectants to medicaments and home fragrances. Especially in cats, even low concentrations of BAC can cause severe symptoms. The outcome has been reported as fatal in 1.2% of cases; in any case, poisoning can result in CNS and respiratory alterations, and painful chemical ulcerations of the skin and mucosae, which can promote secondary infections with prolonged recovery time. Pet owners may not be aware of the serious hazards associated with exposure to BAC-containing products and when an animal presents with signs of oral and/or tongue ulceration with no obvious cause, it is important to question the owner about the recent use of household products that may contain BAC. Finally, this is the first case reported in Italy and we hope that this report will help raise awareness on the hazards associated with BAC products for cats in both domestic and work contexts.

## Data Availability

The datasets used during the current study are available from the corresponding author on reasonable request.

## Abbreviations

BAC: Benzalkonium chloride
QAC: Quaternary ammonium compound
